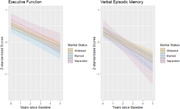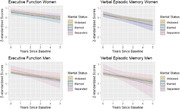# Marital Status and Gender among the Oldest Old: Implications for Cognitive Decline in the Life After 90 Study

**DOI:** 10.1002/alz.092732

**Published:** 2025-01-09

**Authors:** Elisa S Lee, Rachel A. Whitmer, Paola Gilsanz, Claire C. Meunier, Yi Lor, Nancy X Chen, María M. M. Corrada, Alexander Ivan B. Posis, Dan M. Mungas, Charles Decarli, Claudia H. Kawas, Kristen M. George

**Affiliations:** ^1^ University of California, Davis, Davis, CA USA; ^2^ Kaiser Permanente Northern California Division of Research, Oakland, CA USA; ^3^ University of California, Irvine, Irvine, CA USA; ^4^ University of California, Davis School of Medicine, Sacramento, CA USA

## Abstract

**Background:**

Widowhood is associated with cognitive impairment, which is salient for the oldest‐old, who are at high risk of cognitive decline and spousal loss. We assessed the association of marital status with cognitive decline in the LifeAfter90 Study (LA90) and examined effect modification by gender.

**Method:**

LA90 enrolled participants aged 90+ who were long‐term members of Kaiser Permanente Northern California. Analyses included participants identifying as married, separated, or widowed (excluding single and never married individuals due to small numbers). At 6‐month intervals, the Spanish and English Neuropsychological Assessment Scale (SENAS) measured executive function (EF) and verbal episodic memory (VEM), standardized to baseline. Linear mixed models estimated the association of marital status with EF and VEM adjusting for baseline age, race/ethnicity, gender, education, depression, social and emotional support, interview mode and practice effects. Gender stratified models assessed differences between men and women.

**Result:**

Participants (n = 943) had a mean age of 92.4±2.3, 61% were women, 61% were widowed, 29% were married, and 10% were separated. Women were more likely to be separated or widowed. Compared to widows, separated participants had higher average baseline EF (β_EF_ (95% CI):0.14(‐0.07,0.35)) while married participants had lower (β_EF_: ‐0.13(‐0.29,0.03)), though these differences were not statistically significant. Over 1.8±1.35 years of follow‐up, married and separated participants had non‐significant faster decline in EF (Married p_EF_ = 0.33; Separated p_VEM_ = 0.55; Figure 1) and VEM (Married p_EF_ = 0.34; Separated p_VEM_ = 0.07; Figure 1) compared to widows. While associations were not significant in sex‐stratified models, married vs. widowed men had lower baseline EF (β_EF_:‐0.16(‐0.37,0.04)), separated vs. widowed men had better EF (β_EF_:0.29(‐0.19,0.76)), and separated vs. widowed women had better EF (β_EF_:0.12(‐0.13,0.36)). There were no significant differences in decline in sex‐stratified models, though slopes for separated men appeared slightly steeper (Figure 2).

**Conclusion:**

In this diverse cohort of adults ages 90+, there were no significant differences in marital status and cognitive decline, though findings suggested separated participants may average better baseline cognition and steeper decline compared to widowed and married oldest old adults. Further research is necessary to assess the importance of marital status on cognitive aging and decline.